# Mannitol Does Not Enhance Tobramycin Killing of *Pseudomonas aeruginosa* in a Cystic Fibrosis Model System of Biofilm Formation

**DOI:** 10.1371/journal.pone.0141192

**Published:** 2015-10-27

**Authors:** Katherine E. Price, Giulia Orazi, Kathryn L. Ruoff, Wesley P. Hebert, George A. O’Toole, Paul Mastoridis

**Affiliations:** 1 Geisel School of Medicine at Dartmouth, Hanover, New Hampshire, United States of America; 2 Novartis Pharmaceutical Corporation, East Hanover, New Jersey, United States of America; University of Massachusetts Medical School, UNITED STATES

## Abstract

Cystic Fibrosis (CF) is a human genetic disease that results in the accumulation of thick, sticky mucus in the airways, which results in chronic, life-long bacterial biofilm infections that are difficult to clear with antibiotics. *Pseudomonas aeruginosa* lung infection is correlated with worsening lung disease and *P*. *aeruginosa* transitions to an antibiotic tolerant state during chronic infections. Tobramycin is an aminoglycoside currently used to combat lung infections in individuals with CF. While tobramycin is effective at eradicating *P*. *aeruginosa* in the airways of young patients, it is unable to completely clear the chronic *P*. *aeruginosa* infections in older patients. A recent report showed that co-addition of tobramycin and mannitol enhanced killing of *P*. *aeruginosa* grown in vitro as a biofilm on an abiotic surface. Here we employed a model system of bacterial biofilms formed on the surface of CF-derived airway cells to determine if mannitol would enhance the antibacterial activity of tobramycin against *P*. *aeruginosa* grown on a more clinically relevant surface. Using this model system, which allows the growth of robust biofilms with high-level antibiotic tolerance analogous to in vivo biofilms, we were unable to find evidence for enhanced antibacterial activity of tobramycin with the addition of mannitol, supporting the observation that this type of co-treatment failed to reduce the *P*. *aeruginosa* bacterial load in a clinical setting.

## Introduction

Cystic fibrosis (CF) is a human genetic disease caused by mutations in the cystic fibrosis transmembrane conductance regulator gene (CFTR). In the airways, defects in CFTR result in an accumulation of thick sticky mucus, which can become chronically infected with bacterial biofilms that are difficult to clear and are recalcitrant to antibiotic treatment. Of particular concern is infection with the Gram-negative bacterium *Pseudomonas aeruginosa*, as infection with *P*. *aeruginosa* is correlated with CF lung function decline and worsening disease. Lung infections are the primary cause of morbidity and mortality of individuals with cystic fibrosis despite the near-constant administration of antibiotics [1–3]. Therefore, there is a critical need to develop new antibiotics, or alternatively, to develop compounds that render biofilms more sensitive to current therapies.

Several hypotheses explain the antibiotic tolerance of bacterial biofilms including physical barriers to antibiotic penetrance from extracellular matrix that biofilms produce [4, 5], the production of periplasmic glucans [6], slow growth [7] and/or the presence of metabolically inactive persister cells within a biofilm that are inherently tolerant to antibiotics [8–10]. In regard to the persister model, Rice and colleagues recently demonstrated a synergistic effect of tobramycin, the frontline CF maintenance therapy antibiotic, and mannitol versus *P*. *aeruginosa* grown in vitro as a biofilm on an abiotic surface [10]. The authors attribute this increase in tobramycin sensitivity to a stimulation of the persister cells from dormancy as had been previously described for *E*. *coli* [9].

Biofilms formed on abiotic surfaces (plastic or glass), while a relevant model for medical device implants, may not accurately represent the biofilms that form in the CF lung, as they may miss important contributions from the host, including the release of nutrients such as iron and the availability of a biotic substratum to facilitate bacterial colonization [11–14]. In this study, we tested if mannitol treatment would render *P*. *aeruginosa* more sensitive to tobramycin when grown as a biofilm on the surface of airway cells derived from a cystic fibrosis patient homozygous for the ΔF508 allele of CFTR. *P*. *aeruginosa* grown in this model system recapitulates several key aspects of CF lung disease, including robust biofilm formation compared to biofilms formed on non-CF airways cells as well as high-level antibiotic tolerance of these biofilms [12, 13]. Based on our studies, we were unable to find any evidence for enhanced antibacterial activity of tobramycin with mannitol co-treatment on eight strains of *P*. *aeruginosa*, including five CF clinical isolates, using this model system of bacterial biofilm formation on CF airway cells. These results support the observation that a mannitol-tobramycin co-treatment does not reduce bacterial load in patients who have been co-administered [15] and highlight the differences in antibiotic tolerance of biofilms formed on biotic and abiotic surfaces.

## Materials and Methods

### Bacterial strains and growth conditions

Bacterial strains used in this study are listed in Table 1. *P*. *aeruginosa* strains were routinely cultured in lysogeny broth (LB) liquid medium shaking at 37°C or on LB agar incubated at 37°C. Minimum inhibitory concentration (MIC) of tobramycin for *P*. *aeruginosa* strains were measured using Biomerieux E-test strips according to manufacture’s instructions.

**Table 1:** Strains used in this study.

Strain number	Source	Tob MIC (μg/mL)^a^	Mucoid	Reference
*P*. *aeruginosa*				
SMC232	Laboratory strain, *P*. *aeruginosa* PA14	0.75	No	[16]
SMC1585	CF sputum isolate	0.125	Yes	[17]
SMC1587	CF sputum isolate	8.0	No	[17]
SMC1595	CF sputum isolate	1.5	No	[17]
SMC1596	CF sputum isolate	1.0	No	[17]
SMC5450	CF sputum isolate	1.0	Yes	[17]
SMC84	*P*. *aeruginosa* PAO1	0.5	No	[18]
SMC407	*P*. *aeruginosa* FRD1	1.0	Yes	[17, 19]

^a^ Minimum inhibitory concentration of tobramycin for *P*. *aeruginosa* strains as measured by Biomerieux E-test strips according to manufacture’s instructions.

### Tissue culture cultivation

The cystic fibrosis bronchial epithelial (CFBE) cell line used in this study over expresses F508del-CFTR via stable lentiviral transfection of human bronchial epithelial cells [20] (CFBE41o^-^, isolated from a CF patient who was homozygous for ΔF508-CFTR mutation), which were originally immortalized and characterized by Gruenert and colleagues [21, 22]. CFBE cells were the generous gift of J.P. Clancy. IRB approval was not required for the use of this cell line. CFBE cells were cultivated as previously described [12, 13]. Briefly, CFBE cells were seeded into 24-well plates at 50,000 cells/well and fed every other day with minimal essential medium (MEM, Life Technologies) supplemented with 2 mM L-glutamine, 50 U/mL Penicillin, 50 μg/mL Streptomycin, 2μg/mL Puromycin, 5 μg/mL Plasmocin, and 10% Fetal Bovine Serum until confluent and had formed tight junctions [5–7 days).

### Biofilm antibiotic assay on airway cells

Biofilm antibiotic assays were performed as previously described [12, 13]. Briefly, an overnight culture of *P*. *aeruginosa* was washed and resuspended in 1 mL of MEM. *P*. *aeruginosa* inoculum was prepared to an OD_600_ of 0.05 (~5x10^7^ CFUs/mL) in MEM supplemented with 2 mM L-glutamine. Next, 0.5 mL of the *P*. *aeruginosa* suspension was gently added to each well of CFBE cells that had been washed twice with MEM. Cultures were incubated one hour at 37°C, 5% CO_2_. One hour post-inoculation, unattached cells were removed by aspiration and the medium was replaced with MEM supplemented with 2 mM L-glutamine and 0.4% arginine. Six hours post inoculation, the unattached bacteria were removed by aspiration to remove the planktonic fraction of bacteria; the biofilm fraction was washed once with MEM supplemented with 2 mM L-glutamine and 0.4% arginine and incubated with or without 8 μg/mL tobramycin and/or 60 mM mannitol, as indicated, in MEM supplemented with 2 mM L-glutamine and 0.4% arginine. Twenty-one hours post-inoculation, planktonic fractions were removed. MEM was added to wells and the biofilms disrupted by scraping with a pipette tip. The biofilm-grown bacteria were serially diluted and then plated on LB and incubated overnight at 37°C. After overnight incubation, resulting colonies were counted and CFUs were determined. CFUs/well were log_10_ transformed. Log_10_ transformed data was used for all figures and statistical analyses.

### Biofilm antibiotic assay on plastic

An overnight culture of *P*. *aeruginosa* was washed and resuspended in 1 mL of MEM. *P*. *aeruginosa* inoculum was prepared to an OD_600_ of 0.05 (~5x10^7^ CFUs/mL) in MEM supplemented with 2 mM L-glutamine. Next, 0.1 mL of the *P*. *aeruginosa* suspension was gently added to each well of a 96-well culture plate. Cultures were incubated one hour at 37°C, 5% CO_2_. One hour post-inoculation, unattached cells were removed and the medium was replaced with MEM supplemented with 2 mM L-glutamine and 0.4% arginine. Six hours post inoculation, the unattached bacteria were removed and the biofilm fraction was incubated with or without 80 μg/mL tobramycin and/or 60 mM mannitol in MEM supplemented with 2 mM L-glutamine and 0.4% arginine. Twenty-one hours post-inoculation, planktonic fractions were removed. MEM was added to wells and biofilms were disrupted using a solid pin multi-blot replicator. The biofilm-grown bacteria were serially diluted and then plated on LB and incubated overnight at 37°C. After overnight incubation, resulting colonies were counted and CFUs were determined. CFUs/well were log_10_ transformed. Log_10_ transformed data was used for all figures and statistical analyses.

### Cytotoxicity Assay

Cytotoxicity was measured as fraction of lactate dehydrogenase (LDH) release using the CytoTox 96 Non-radioactive Cytotoxicity Kit (Promega) according to the manufacturer’s instructions. CFBE cells were incubated with 0, 40 or 60 mM mannitol (dissolved in MEM) for 24 hours at 37°C, 5% CO_2_. Supernatants were collected and used in the CytoTox 96 Non-radioactive Cytotoxicity Kit. Cells treated with Triton X-100 detergent served as the total lysis control. Fraction cytotoxicity was determined by dividing the absorbance at 450 nm of each sample (OD_450_) by the OD_450_ of the total lysis control.

## Results

### Mannitol co-treatment does not increase the susceptibility of a laboratory strain of *P*. *aeruginosa* to tobramycin

**Fig 1:**
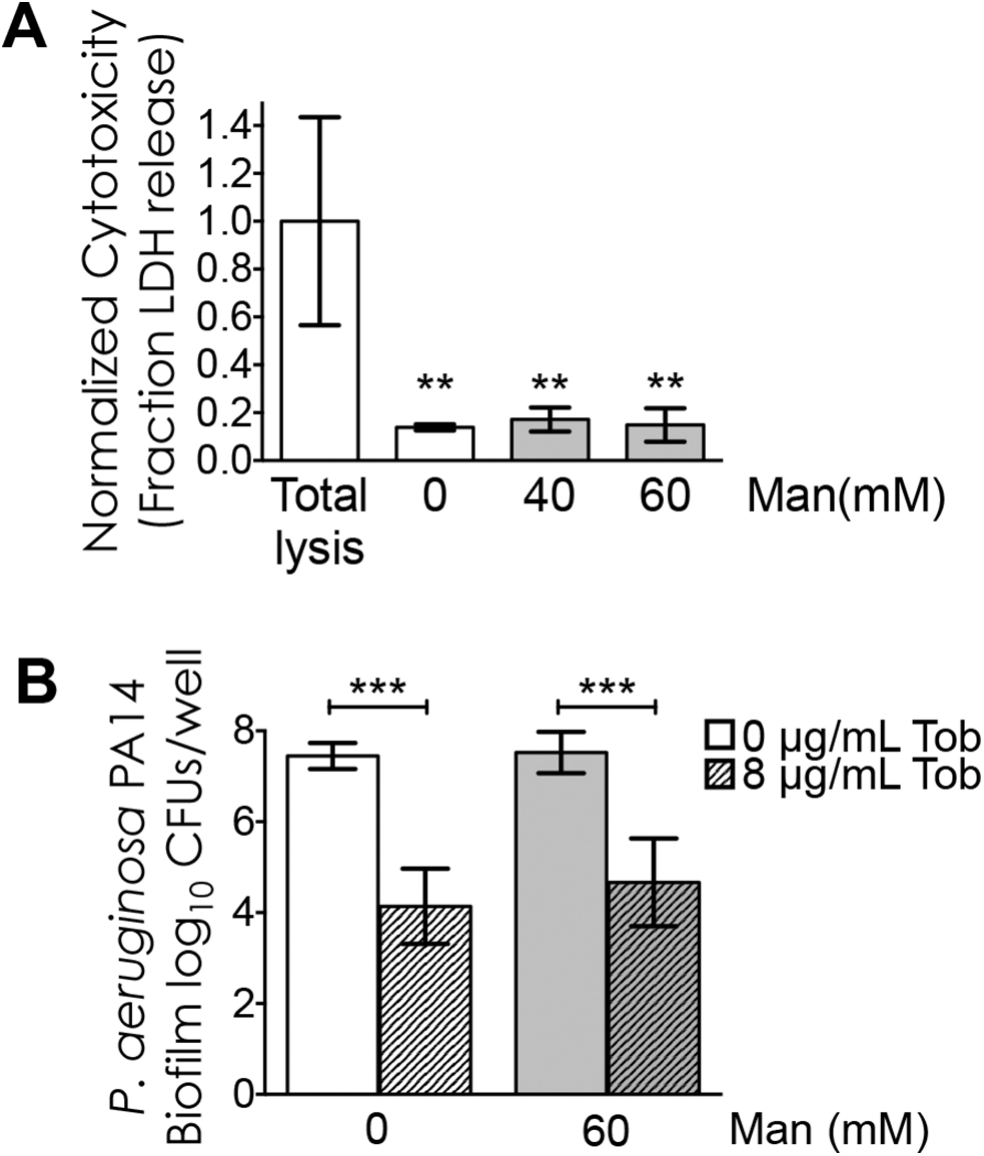
Mannitol does not sensitize non-mucoid, laboratory strain *P*. *aeruginosa* PA14 to tobramycin. A. Mannitol is minimally cytotoxic to CFBE cells. Normalized cytotoxicity as measured by fraction of LDH release. Cytotoxicity was measured after 24 hours of treatment with 0, 40 or 60 mM mannitol as indicated. Cells lysed with Triton X-100 served as a control to determine total lysis. Columns indicate mean of at least three biological replicates, error bars indicate standard deviation (S.D.). **, P<0.01, comparison of indicated sample to total lysis control by ordinary one-way ANOVA with Tukey’s post test for multiple comparisons. B. Viability of *P*. *aeruginosa* PA14 grown as a biofilm on CFBE cells after treatment with 0 μg/mL tobramycin (open bars), 8 μg/mL tobramycin (hatched bars), 0 mM mannitol (white bars), 60 mM mannitol (gray bars) or co-treatment with 8 μg/mL tobramycin and 60 mM mannitol, as indicated. Columns indicate mean of at least three biological replicates, error bars indicate S.D. ***, P<0.001 by ordinary one-way ANOVA with Tukey’s post test for multiple comparisons. There is no significant difference between *P*. *aeruginosa* PA14 treated with tobramycin +/- mannitol.

To test the hypothesis that mannitol would increase the antibacterial activity of tobramycin on CF airway cell-grown biofilms of *P*. *aeruginosa*, we first measured the cytotoxic effect of mannitol on CF airways cells to ensure that the dose of mannitol used in subsequent studies did not damage the host cells. In these studies, CF-derived bronchial epithelial (CFBE) cells, which are homozygous for the ΔF508 mutation in CFTR, were used as the substratum to grow the *P*. *aeruginosa* biofilms. To assess cytotoxicity of mannitol, CFBE cells were incubated with increasing concentrations of mannitol, or medium alone as a control, for 24 hours. Supernatants were collected, bacteria were removed by centrifugation and clarified supernatants were used to determine cytotoxicity, which was expressed as the fraction of the cytoplasmic enzyme LDH released, using a colorimetric assay. LDH activity was normalized to a total lysis control, set at 1.0, wherein the host cells were treated with Triton-X 100, a detergent used to burst cells open. Treatment with 40 mM or 60 mM mannitol, which falls within the range of concentrations used in previous studies with abiotic biofilms [10], resulted in low cytotoxicity (less than 20% of total lysis), comparable to treatment with tissue culture medium alone (Fig 1A). Because the higher dose had minimal cytotoxicity, we used 60 mM mannitol in all subsequent assays.

To test if mannitol treatment could sensitize *P*. *aeruginosa* to tobramycin treatment in the context of a biofilm formed on the surface of airway cells, we established *P*. *aeruginosa* strain PA14 biofilms on the surface of CF airway cells as previously described [12, 13]. Biofilms were established for six hours before treatment with medium, tobramycin, mannitol or co-treatment with tobramycin and mannitol. After fifteen hours of treatment, the viability of *P*. *aeruginosa* growing as a biofilm was determined by colony counts. Treatment with 60 mM mannitol alone resulted in similar viability to treatment with medium alone. Treatment with 8 μg/mL of tobramycin resulted in a 3.3-log_10_ reduction in viability of biofilm grown bacteria. Co-treatment of *P*. *aeruginosa* grown as a biofilm on CFBE cells with 8 μg/mL of tobramycin and 60 mM mannitol resulted in a 2.9-log_10_ reduction in viability; a difference which is not statistically significant from treatment with tobramycin alone, suggesting that mannitol does not sensitize *P*. *aeruginosa* to tobramycin (Fig 1B).

### Mannitol co-treatment does not increase the susceptibility of CF clinical isolates of *P*. *aeruginosa* to tobramycin

The laboratory strain *P*. *aeruginosa* PA14, while a recent isolate of this microbe, was obtained from a burn wound and may not accurately reflect the strains of *P*. *aeruginosa* found in the airways of individuals with CF. We therefore examined the effects of mannitol co-treatment with tobramycin on 5 clinical strains isolated from sputum of individuals with CF (Table 1). These strains exhibited a range of tobramycin resistance and mucoidy as previously characterized [17]. Strains SMC1585 and 5450 are mucoid, while strains SMC1587, 1595 and 1596 are non-mucoid; the tobramycin MIC for planktonically grown cultures of these strains is shown in Table 1.

**Fig 2:**
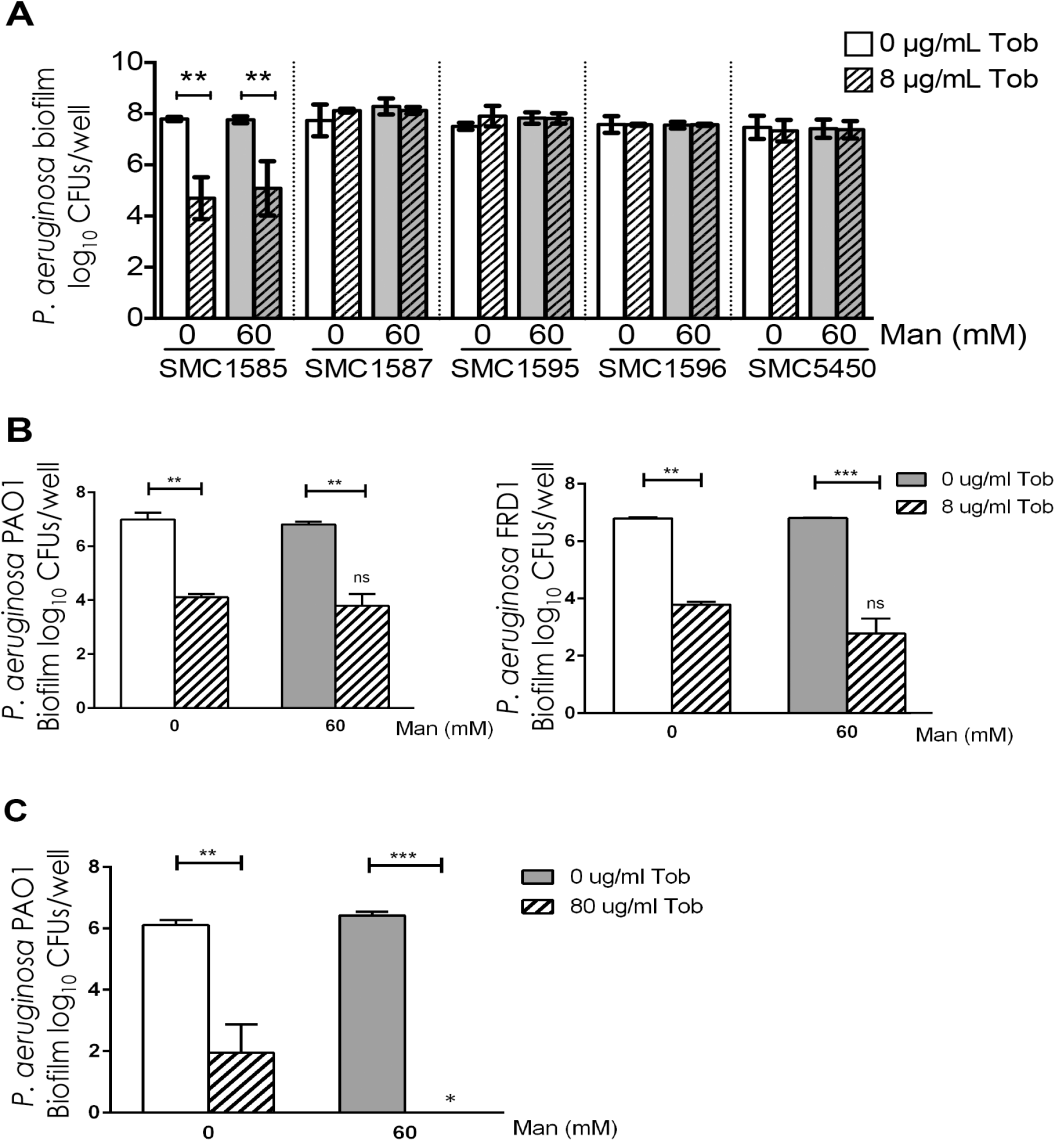
Mannitol does not sensitize *P*. *aeruginosa* clinical isolates grown as biofilms on CF airway cells to tobramycin. A. Viability of *P*. *aeruginosa* clinical isolates grown as biofilms on CFBE cells and treated with 0 μg/mL tobramycin (open bars), 8 μg/mL tobramycin (hatched bars), 0 mM mannitol (white bars), 60 mM mannitol (gray bars) or co-treatment with 8 μg/mL tobramycin and 60 mM mannitol, as indicated. Columns indicate mean of at least three biological replicates, error bars indicate S.D. **, P<0.01 by ordinary one-way ANOVA with Tukey’s post test for multiple comparisons. B. The viability of strains *P*. *aeruginosa* PAO1 (left) and FRD1 (right) as biofilms on CFBE cells and treated with 0 μg/mL tobramycin (open bars), 8 μg/mL tobramycin (hatched bars), 0 mM mannitol (white bars), 60 mM mannitol (gray bars) or co-treatment with 8 μg/mL tobramycin and 60 mM mannitol, as indicated. **, P<0.01 or ***, P<0.001 by ordinary one-way ANOVA with Tukey’s post test for multiple comparisons. ns, not significant compared to tobramycin treatment in the absence of mannitol. C. The viability of strain *P*. *aeruginosa* PAO1 as a biofilm on plastic and treated with 0 μg/mL tobramycin (open bars), 80 μg/mL tobramycin (hatched bars), 0 mM mannitol (white bars), 60 mM mannitol (gray bars) or co-treatment with 80 μg/mL tobramycin and 60 mM mannitol, as indicated. *, P<0.05 compared to treatment with 80 μg/mL tobramycin with no mannitol. **, P<0.01 or ***, P<0.001 by ordinary one-way ANOVA with Tukey’s post test for multiple comparisons.

There was no reduction of *P*. *aeruginosa* viability from mannitol treatment alone compared to treatment with medium alone for any of the clinical isolates tested (Fig 2). Additionally, there was no reduction of *P*. *aeruginosa* viability in biofilms co-treated with mannitol and tobramycin (at 8 μg/ml, the same concentration tested for the laboratory isolate) compared to treatment with tobramycin alone (Fig 2A). These results suggest that mannitol does not sensitize *P*. *aeruginosa* isolated from the airways of individuals with CF to tobramycin when grown as a biofilm on CF airway cells.

As an additional control, we also investigated the impact of mannitol on biofilms of *P*. *aeruginosa* PAO1 (a lab strain) and *P*. *aeruginosa* FRD1 (a clinical mucoid strain) treated with tobramycin. Mannitol did not enhance tobramycin-mediated killing of either of these strains (Fig 2B). Finally, we replicated the original finding of Rice and colleagues [10]. As was originally reported, when the biofilm of *P*. *aeruginosa* PAO1 was grown on plastic, the addition of mannitol enhanced tobramycin-mediated killing by ~100-fold (Fig 2C). It is important to note that while in our experiments we saw substantially more killing of *P*. *aeruginosa* PAO1 treated with tobramycin alone than was observed by Rice, et al. [10] we still observed the originally reported mannitol-enhanced killing.

## Discussion

The bacteria that chronically infect the lungs of individuals with CF are recalcitrant to antibiotics. One hypothesis for this tolerance to treatment is that the bacteria are in a metabolically dormant state and are therefore intrinsically resistant to antibiotic pressure [8]. Because this tolerance is not genetically encoded, if these cells could be brought out of dormancy, they would then be sensitive to antibiotic killing. Such examples have been shown in *E*. *coli* [9] and in *P*. *aeruginosa* [10]. In the case of *P*. *aeruginosa*, a sub-set of strains of this microbe (strains PAO1 and FRD) grown as biofilms formed on a abiotic (plastic) surface were approximately 1000-fold more sensitive to tobramycin treatment with co-administration of mannitol and tobramycin. We replicated that finding here with a biofilm of *P*. *aeruginosa* PAO1 grown on plastic. However, an additional clinical isolate 18A was resistant to tobramycin, and co-administration of mannitol and tobramycin did not sensitize this strain, suggesting that tobramycin sensitization by mannitol is strain specific and is not generalizable.

To address whether the synergy of mannitol and tobramycin might be relevant to a CF patient population, we used our in vitro model system of bacterial biofilm formation on CF airway cells to assess whether this synergy also occurs on a more clinically relevant surface. *P*. *aeruginosa* grown in our model system recapitulate several key aspects of chronic biofilm formation, including formation of biofilm-like microcolonies, expression of genes associated with biofilm growth, induction of quorum sensing, the requirement for genes necessary for biofilm formation on abiotic surfaces and, of clinical importance, high-level antibiotic tolerance consistent with biofilms in clinical settings [12, 13]. Using this system, we were unable to recapitulate the sensitization effect that mannitol gave to tobramycin treated biofilms grown on plastic. This absence of synergy held true for the lab strains *P*. *aeruginosa* PA14 and PAO1, the well-characterized mucoid clinical isolate *P*. *aeruginosa* FRD1 as well as five other clinical strains of *P*. *aeruginosa* isolated from individuals with CF, including strains showing planktonic tobramycin resistance as well as mucoid and non-mucoid strains.

Mannitol is currently utilized as a therapeutic that hydrates mucus allowing for increased mucociliary and cough clearance of retained secretions in the airways of individuals with CF [15, 23]. Our model system does not produce mucus, therefore we cannot test the effect that mucus viscosity/ hydration has on tobramycin efficacy. It is formally possible that better hydration of the airways may allow better penetrance and therefore better effectiveness of antibiotics. However, in the clinical trial, no change in *P*. *aeruginosa* load was observed in patients given mannitol versus placebo control despite maintaining their current CF therapies including inhaled antibiotics [15], supporting our findings that tobramycin and mannitol do not synergize in the treatment of *P*. *aeruginosa* biofilms formed on airway cells.

Experiments presented here suggest that the mechanism of action of mannitol via stimulation of *P*. *aeruginosa* out of a dormant state and into metabolically active one that is sensitive to tobramycin, as suggested by Rice *et al*. [10], is unlikely to be occurring in biofilms formed on biotic surfaces. Additionally, our results illustrate that biofilms grown on biotic surfaces are distinct from biofilms grown on plastic, as has been previously reported [11–13] and highlights the cost and time savings achieved by using biofilms formed on biotic surfaces as an important tool in preclinical studies for drug discovery.
